# A Comprehensive study of the Effects of Chain Morphology on the Transport Properties of Amorphous Polymer Films

**DOI:** 10.1038/srep29092

**Published:** 2016-07-11

**Authors:** Dan Mendels, Nir Tessler

**Affiliations:** 1 The Sarah and Moshe Zisapel nanoelectronic center, Electrical Engineering Dept., Technion Israel institute of technology, Haifa 32000, Israel

## Abstract

Organic semiconductors constitute one of the main components underlying present-day paradigm shifting optoelectronic applications. Among them, polymer based semiconductors are deemed particularly favorable due to their natural compatibility with low-cost device fabrication techniques. In light of recent advances in the syntheses of these classes of materials, yielding systems exhibiting charge mobilities comparable with those found in organic crystals, a comprehensive study of their charge transport properties is presented. Among a plethora of effects arising from these systems morphological and non morphological attributes, it is shown that a favorable presence of several of these attributes, including that of rapid on-chain carrier propagation and the presence of elongated conjugation segments, can lead to an enhancement of the system’s mobility by more than 5 orders of magnitude with respect to ‘standard’ amorphous organic semiconductors. New insight for the formulation of new engineering strategies for next generation polymer based semiconductors is thus gathered.

Recent realizations of disordered polymer films characterized by high charge carrier mobilities have triggered considerable interest in light of their unexpected conducting qualities and their consequent technological potential^1^,^2^,^3^,^4^,^5^,^6^. Reasoning for these systems’ high mobility values has been pointed towards their morphological attributes and particularly the presence of rapid charge propagation along the polymer chains’ backbones, as has been observed via spectroscopic techniques^7^. In semi-crystalline materials, thus, the observed high mobilities have been conjectured to arise from rapid carrier propagation through interconnected (by polymer chains) crystalline regions^1^,^8^,^9^ while in amorphous materials, from the presence of the rapid on-chain carrier propagation itself^2^,^3^. Additional causes for these materials’ high mobilities have also been shown to potentially arise from their exceptionally low inherent energetic disorder^4^,^10^ and the short lifetimes of the carrier traps they consist^10^.

In the present study, to gain insight regarding the importance of chain morphology on the transport properties of amorphous polymer films, Monte Carlo transport simulations were implemented on artificially constructed films. The simulations’ implementation was based on the Gaussian Disorder Model (GDM) framework given its predominant role as a modeling tool for transport in disordered organic semiconductors. This in turn allowed to carry out meaningful comparisons between the study’s findings with outputs obtained from standard implementations of the GDM framework^11^,^12^,^13^,^14^, thus, facilitating their discernment. Employing the GDM allowed also the examination of the system over a wide range of sets of parameters. In this respect the presented study can, thus, be viewed as complementary to ab-intio studies. These tend to focus on particular material structures and their specific microscopic attributes^4^,^5^,^10^,^15^ but by nature deal less with the effects of these attributes over ranges of parameter values.

## Results

To implement a charge transport model that stems from the GDM framework, polymer chain segment configurations were constructed on three dimensional cubic lattices by implementing customized variations of the Slithering Snake algorithm^16^,^17^ following a similar procedure put forward in ref. 18. Chains of length *L*, representing polymer segments over which continues rapid carrier propagation can occur (but considered to be weakly electronically coupled to each other^19^), were placed within a base lattice of lattice constant *a*, where a filling factor *ρ* was maintained and for which periodic boundary conditions were defined.

**Figure 1:**
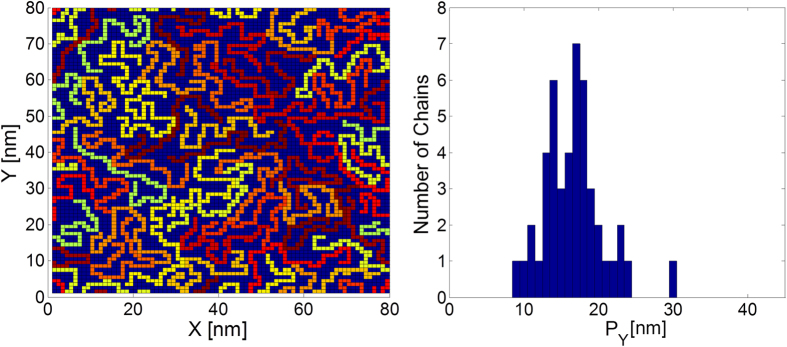
Left: A two dimensional lattice configuration obtained from a custom modified Slithering Snake simulation, implemented on a cubic lattice of size 80 *nm* × 80 *nm*, with chains’ length *L* = 60 *nm* and a chain filling factor *ρ* = 0.43. The simulation was initially run at *T* = 10^4 ^*K* with Δ*E*_inter_ = 0.02 *eV*, Δ*E*_intra_ = 1.7 *eV* and 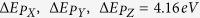. Upon reaching steady-state the simulation was cooled to *T* = 150 °*K* and run until steady-state was reached again with Δ*E*_inter_ = 0.02 *eV*, Δ*E*_intra_ = 0.04 *eV* and 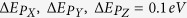. Right: The obtained LC’s distribution of chain projections on the 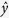 axis.

To study the influence of the specified system morphological attributes within an energetically disordered system, lattice configurations (LCs) characterized by a wide range of parameter values were constructed using the methodology described in the *Methods* section (a list of the LCs for which results are presented in the article is provided in Table 1). While all the transport calculations were implemented on 3D base-lattices, to illustrate the form of the constructed LCs, a two dimensional lattice configuration obtained from a simulation implemented on a base-lattice of size 80 *nm* × 80 *nm* with chains’ length *L* = 60 *nm* and a filling factor *ρ* = 0.43 is presented in Fig. 1. Presented along, is the LC’s distribution of chain projections *P*_*Y*_ on the 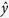 axis.

While employing the Slithering Snake algorithm was found to provide flexibility in the construction of the LCs, LCs with an average number of nearest neighboring chain-sites that is above ~3 were difficult to attain (the average number of neighbors per site for a cubic lattice is 6). We found that this low limit was due to the maximum density of chains the algorithm could cope with^20^. Given the importance of this morphological attribute and to allow for higher neighboring statistics and increase the range of parameter values which can be explored in this context, a distance reduction rule (described in the *Methods* section) for calculating effective distances between LC sites was devised.

Charge transport Monte Carlo simulations (MCs) were implemented on the attained LCs, in similarity with standard simulations of the GDM^11^,^13^,^21^,^22^ with the distinct difference that carriers were allowed to visit only chain sites as opposed to vacant base-lattice sites. As within simulations implemented under the premise of the GDM framework, each chain site in the simulations represented a localized electronic state and was assigned an energy drawn randomly from the system density of states (DOS) which was taken to be in the form of a Gaussian,


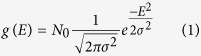


where in Eq. (1), *σ* represents the DOS standard deviation and *N*_0_ the number of states per unit volume in the system. To enable a comparison between the system’s transport properties under the premise of bare charge transport and polaron transport, both Miller-Abrahams^23^ and Marcus-theory^24^ hopping rate expressions were used, Eqs (2 and 3) respectively (see *Methods*). More general expressions^25^ are outside the scope of the current paper.

The remainder of the results section is, thus, organized as follows. *Section 1* examines the effects of neighboring statistics and rapid on-chain carrier propagation on the system’s transport and thermoelectric properties. *Section 2* explores the influence of the chains’ length, their average projection on the field axis and the ratio between the two on the system’s transport properties. An exploration of the influence of the degree of energetic disorder on the transport properties of amorphous polymer films is presented in *Section 3*. Finally, *Section 4* investigates the influence of the polymer-chains’ conjugation-segments’ length and the charge transport mechanism on the transport properties of these systems as well.

### Section 1–Neighboring Statistics

**Figure 2:**
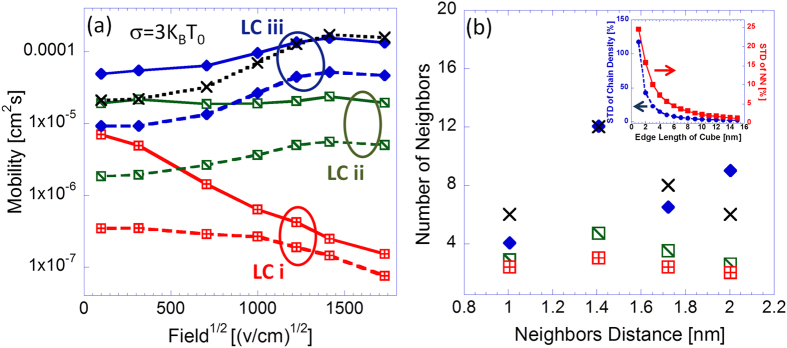
(**a**) Mobility field dependence curves obtained from simulations implemented on LCs characterized by the neighboring statistics shown in (**b**). In (crossed-boxes) data obtained from LC i which fill factor is *ρ* = 0.22, in (diagonalised boxes) data obtained from simulations implemented on LC ii which fill factor is *ρ* = 0.42 and in (diamonds) data obtained from simulations implemented on LC iii which fill factor is *ρ* = 0.42 and for which the distance reduction rule was applied. In dashed lines data obtained from simulations in which 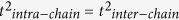 and in solid lines data obtained from simulations in which 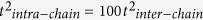, i.e. in the latter rapid on-chain carrier propagation was incorporated. In (Xs), data obtained from simulations implemented on a standard cubic lattice. All results were obtained from simulations implemented with a Gaussian DOS with a standard deviation *σ* = 3 *K*_*B*_*T*_0_ where *T*_0_ = 300 °*K* and with temperature *T* = 300 °*K* and *n* = 6 × 10^−5^ carriers per chain site in the system. Inset of (**b**): typical profiles (taken from LC iii) of the standard deviations of the average number of chain sites (circles) and average number of nearest neighboring sites per site (sqaures), each divided by the corresponding average LC value, as function of z (sub-lattice edge length).

One of the important morphological attributes of amorphous polymer films focused on in this study is its neighboring statistics, i.e. the average number of neighboring chain states each chain state in the system has at any given distance. These statistics are largely controlled by the system chain density (quantified in this article by the system’s fill factor), but are also influenced by the system’s degree of homogeneity and other more subtle morphological attributes. Figure 2 compares results obtained from MC transport simulations implemented on three different LCs, the first corresponding to a fill factor of *ρ* = 0.22 (LC i), the second of *ρ* = 0.42 (LC ii) and the third of *ρ* = 0.42 (LC iii) as well but to which the distance reduction rule was applied. All three LCs were constructed on a base lattice of size 140 *nm* × 140 *nm* × 140 *nm* with 90 *nm* long chains and average chain projections on the system axes of *P*_*X*,*Y*,*Z*_ ≈ 30 *nm* (the systems’ grid has 1 nm spacing).

To ensure that the utilized LCs were spatially homogenous at scales beyond several nano-meters, the following procedure was implemented. LCs were divided into boxes of size *z*^3^ and the standard deviations of the average number of chain sites and average number of nearest neighboring chain sites per chain site in a box were computed as function of *z*. Typical profiles obtained from this procedure are presented in the inset of Fig. 2b, where the ratios between the measured standard deviations and the corresponding average LC values are observed to fall beneath 10% for boxes with edges longer than *z* = 4 *nm*. Namely on a scale larger than 4 nm the represented samples were practically uniform.

The mobility field dependence obtained from transport simulations implemented on LCs i, ii and iii is presented in Fig. 2a, where the respective neighboring statistics of the utilized LCs are presented in Fig. 2b. Results are shown both for simulations in which no rapid on-chain carrier propagation was incorporated (dotted-dashed lines and empty symbols) and for those in which it was (solid lines and filled symbols). For the latter, the transfer integrals affiliated with intra-chain sequential sites were set to equal ten times those affiliated with inter-chain nearest neighbors (NN) 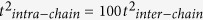, namely the energy independent component of Eq. (2) was set to be 100 times larger for intra-chain hops (between sequential sites) than for inter-chain ones. For reference, results obtained from corresponding simulations implemented on a standard cubic lattice (Xs) are presented as well. We start by examining the results obtained from simulations in which no rapid on-chain carrier propagation was implemented, namely the role of the polymer chains in this case was only in dictating the spatial distribution of the carrier sites. These are shown as dashed lines and empty symbols in Fig. 2a. As one moves from LC iii to ii to i and the systems’ neighboring statistics is lowered (shown in Fig. 2b), the corresponding mobility values as well as the concomitant field dependence slopes are observed to decrease. Adding the presence of on-chain carrier propagation (solid lines and filled symbols) and comparing the obtained results with the corresponding non-rapid on-chain carrier propagation results, an increase of the mobility values can be observed, but also a decrease of the concomitant field dependence slopes can be observed as well.

Discerning the described effects, the increase of the system’s carrier mobility with the neighboring statistics seems intuitive. Namely, increasing the latter translates to an increased number of chain intersections (i.e. sites from which carriers can hop to other chains’ sites), enabling carriers to traverse via more efficient paths through the system. Similarly, the introduction of rapid on-chain propagation enables fast carrier transport over increased distances along the chains and more frequent carrier chain intersection visitations. In contrast, as the system neighboring statistics is lowered, an increase of the applied field is more prone to induce carrier trapping at chain ends and bends, thereby leading to the observed decrease of the mobility field dependence slopes. Similarly, the presence of on-chain carrier propagation reduces the likelihood of inter-chain hopping, thereby contributing to the field induced trapping effect.

**Figure 3:**
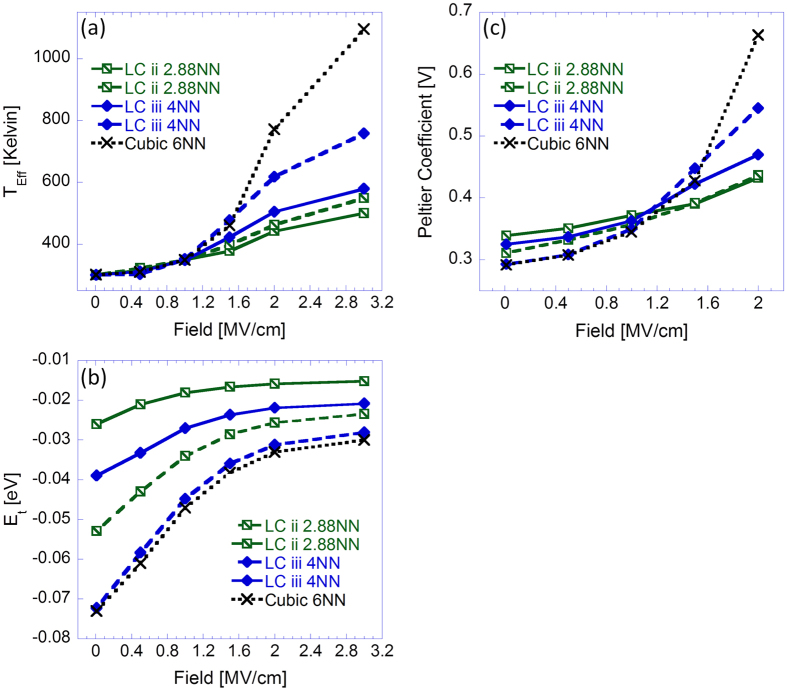
(**a**) Carriers’ effective temperature, (**b**) transport energy and (**c**) Peltier coefficient as function of applied field obtained from simulations implemented on LCs ii (diagonalised squares) and iii (diamonds). In dashed lines data obtained from simulations in which 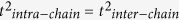 and in solid lines data obtained from simulations in which 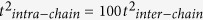, i.e. in the latter rapid on-chain carrier propagation was incorporated. In (Xs) data obtained from transport simulations implemented on a cubic lattice. All results were obtained from simulations implemented with a Gaussian DOS with a standard deviation *σ* = 3 *K*_*B*_*T*_0_ (where *T*_0_ = 300 °*K*), with temperature *T* = 300 °*K* and *n* = 6 × 10^−5^ carriers per chain site in the system.

To farther illustrate the importance of the system’s neighboring statistics, we examine the thermoelectric effect related carrier effective temperature *T*_*eff*_ , transport energy *E*_*t*_, and Peltier coefficient as function of the applied field, all measured using the methodologies presented in refs 13,14,26,27. Figure 3 presents the results obtained from simulations implemented on LCs ii and iii as well as for simulations implemented on a cubic lattice. The effective temperature field dependence results are presented in Fig. 3a whereas the transport energy and Peltier coefficient field dependences are presented in Fig. 3b,c, respectively.

Comparing the results obtained from standard GDM simulations (X symbols) to those obtained from simulations implemented on LCs ii and iii, we find that carrier heating is suppressed due to the reduction of the system’s neighboring statistics and to greater extent the incorporation of rapid on-chain carrier propagation in the simulations (Fig. 3a). To understand both effects we need first to realize that carrier heating takes place when the applied electric field makes higher energy states more accessible for (down field) hopping and consequently shortens carrier dwell times at lower energies^26^,^28^. Within a polymer film like system, however, and to a greater extent in one in which rapid on-chain propagation is incorporated, this effect is suppressed due to the carrier inclination (or full limitation in the case of a low density system) to propagate along the chains, thereby limiting the described field induced effects. In other words, since carriers are inclined or even forced to hop along chains (more often than not in directions perpendicular to the applied field), field induced heating substantially decreases.

For the same reason carriers become increasingly limited in their ability to optimize their paths in energy space^22^ as the system’s neighboring statistics is reduced or rapid on-chain carrier propagation is incorporated. Consequently, carriers visit site energies located at the center of the system’s DOS (where the site density is highest) at higher frequencies, thereby leading to an upward shift of the system’s transport energy (Fig. 3b), i.e. the average visited site-energy.

Upon obtaining the system’s transport energy and carrier occupation distributions (used for computing *T*_*eff*_), the system’s Peltier coefficient Π (Fig. 3c) can be calculated via 
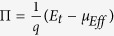
29,30 and 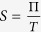
31 (for low fields), respectively. Here, *μ*_*Eff*_ is the carrier effective quasi-chemical potential^14^. Using these relations we have found that due to higher transport energy values at low fields, the polymer film like morphologies give rise to Peltier coefficient and Seebeck coefficient values higher by 10–20% than those obtained for a standard cubic lattice. In contrast, as carrier heating takes effect at high fields, causing the reduction of *μ*_*Eff*_^14^, the field dependent Peltier coefficient obtained from the polymer film like systems consequently becomes substantially smaller than that corresponding to the cubic lattice system.

Having established that the neighboring statistics is a crucial system attribute, we move on to study the effects of other morphology related parameters while keeping this statistics fixed. To make the effect of the other parameters more apparent we choose the neighboring statistics that is closest to the standard GDM model, namely the distance reduction rule is applied.

### Section 2–Chain Length, Chain Projections on the System Axes and Chain Rigidity

**Figure 4:**
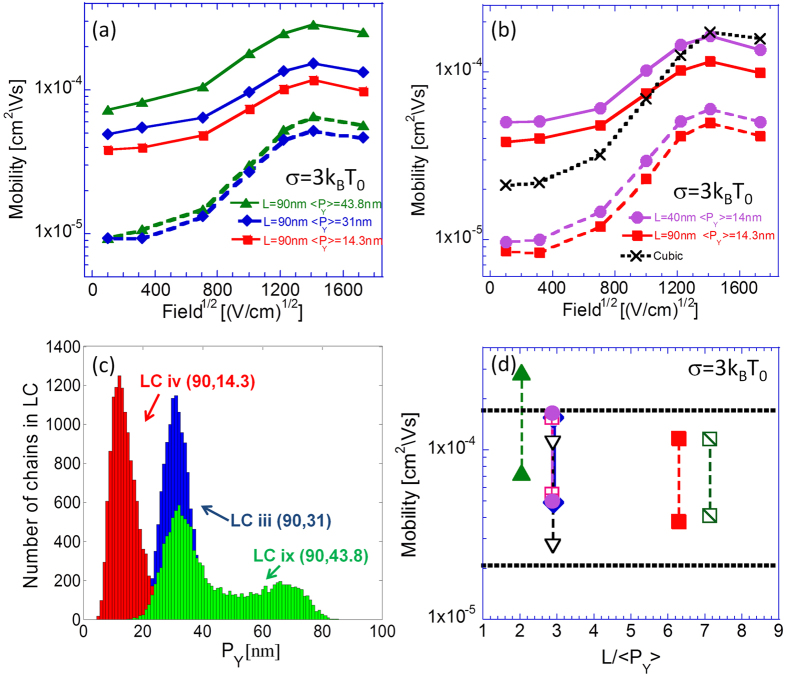
(**a**) Mobility field dependence curves obtained for fixed chains’ length and different projections in the field direction 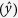. (**b**) Comparison between mobility field dependence curves obtained from LCs with similar average projections but different chain lengths (40 nm & 90 nm). For comparison we also added the results of a cubic lattice (not polymers). In (**a**,**b**) dashed lines represent data obtained from simulations in which 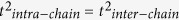 and solid lines data obtained from simulations in which 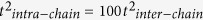. All transport results were obtained from simulations implemented with a Gaussian DOS with a standard deviation *σ* = 3 *K*_*B*_*T*_0_ (*T*_0_ = 300 °*K*) at temperature *T* = 300 °*K* and *n* = 6 × 10^−5^ per chain site in the system. (**c**) Chain projection distributions *P*_*Y*_ obtained from LCs iii, iv and ix. (**d**) Low field mobilities *F* = 10^4 ^*Vcm*^−1^ and high field mobilities *F* = 2 ⋅ 10^6 ^*Vcm*^−1^ taken from simulations in which rapid on-chain carrier propagation was incorporated as function of the systems’ *L*/〈*P*_*Y*_〉 ratio. Horizontal dotted lines represent the corresponding values obtained from simulations implemented on a cubic lattice. Symbols: data corresponding to (chain length [nm], projection [nm]) of–(90,31) in diamonds (LC iii), (90,14.3) in squares (LC iv), (140,19.6) in diagonalized squares (LC v), (140,49) in crossed squares (LC vi), (40,14) in circles (LC vii), (10,3.45) empty triangles (LC viii), (90,43.8) filled triangles (LC ix) and cubic lattice (Xs).

In the present section we investigate the influence of the system’s chains’ length, their average projections on the field axis, and the ratio between these two parameters. Since in all simulations but one the chains were isotropically oriented in space (〈*P*_*X*_〉 = 〈*P*_*Y*_〉 = 〈*P*_*Z*_〉), the latter ratio was predominantly determined by the profusion of bends and twists within the chains, and hence can be regarded as a measure of the chains’ rigidity^2^,^3^. As in the previous section, the incorporation of rapid on-chain carrier propagation is examined as well. LCs corresponding to a range of the described parameters’ values were constructed with a filling factor *ρ* = 0.42, spatial homogeneity profiles similar to those presented in the inset of Fig. 2b and neighboring statistics similar to that of LC iii (Diamonds in Fig. 2b). All used LCs were isotropic and were characterized by Gaussian projection distributions, LC ix being the exception as shown in Fig. 4d.

The present section’s analysis began with the observation of Fig. 4a in which a comparison of mobility field dependence curves obtained from simulations of LCs incorporating the same chains’ length but different average chain projections 〈*P*_*Y*_〉 is presented, with 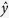 being the direction of the applied filed. The chains’ length was chosen as 90 nm and the projections on the field direction were 14.3 nm (squares, LC iv), 31 nm (diamonds, LC iii), and 43.8 nm (triangles, LC ix). The latter, LC ix, was run while adding some preference to chain alignment in the field direction (this is the only lattice that is not isotropic).

Figure 4b similarly presents a comparison between mobility field dependence curves obtained from simulations implemented on LCs incorporating similar average chain projections 〈*P*_*Y*_〉 ≈ 14 *nm*, but different chains’ length. The 40 nm long (LC vii) is denoted with circles and the 90 nm long (LC iv) with squares. For reference we added results obtained from simulations implemented on a cubic lattice which corresponds to the standard GDM model (X symbols).

Figure 4c shows chain projection distributions with those of LC iv and LC iii showing a Gaussian like distribution which is typical for the isotropic equilibrium state. The average projection of LC iv is smaller than that of LC iii since the former is more entangled. This was achieved by setting Δ*E*_*Pi*_ to zero for LC iv instead of the Δ*E*_*Pi*_ = 0.1 *eV* used for LC iii (see Table 1). The shape of the distribution of LC iv is different and has two peaks. This is a result of the structural simulation being stopped before reaching the equilibrium state. Since the initial state is with all chains aligned in the field direction some of the chains remained preferentially along that direction (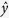).

Observing the mobility field dependence curves obtained from simulations in which no rapid on-chain carrier propagation was incorporated (dashed lines in sub-Fig. 4a,b), an insensitivity to the chains’ lengths and their average projection values is found. Interestingly, the mobility field dependence curves obtained from simulations in which rapid on-chain carrier propagation was incorporated (solid lines) seem to display only a weak dependence on the LCs’ average chain projections and chains’ length. Thus for example, increasing 〈*P*_*Y*_〉 by a factor of ~2 while keeping the system’s chains’ length constant leads only to a ~20% increase in the system’s mobility (Fig. 4a) whereas, increasing the chains’ length by roughly a factor of 2 while keeping 〈*P*_*Y*_〉 constant leads to the lowering of the system mobility by ~20% (Fig. 4b).

We find that the, nevertheless, observed moderate differences between the mobility field dependence curves can be attributed to two main factors. The first, is simply the average chain projection on the field axis. As it increases, carriers can propagate over larger net distances through the system without needing to hop between chains^2^,^3^. The second is the ratio between the chains’ length and their average projections *L*/〈*P*_*i*_〉which is equivalent to the chains’ rigidity in the case in which the LCs are isotropic. Its effect can be clearly observed in Fig. 4b in which mobility values corresponding to LC vii (*L* = 40 *nm*; 〈*P*_*Y*_〉 = 14 *nm*, *circles*) can be seen to be higher than those corresponding to LC iv (*L* = 90 *nm*; 〈*P*_*Y*_〉 = 14.3 *nm*, *squares*) despite the chains within the latter being more than twice as long. Indeed a negative correlation seems to arise between the chains’ *L*/〈*P*_*Y*_〉 ratio and the system’s mobility as illustrated in Fig. 4d. We find that the negative correlation can be attributed to the increasing number of chain bends within the system (which can act as traps) as the system’s *L*/〈*P*_*Y*_〉 ratio rises^2^,^3^. Furthermore, a larger *L*/〈*P*_*Y*_〉 ratio can allude to more chain self-entanglement and, thus, to a diminished inter-chain interface in the system. Namely, increasing *L*/〈*P*_*Y*_〉 leads to a reduction of the carriers’ ability to exercise inter-chains transitions.

In Fig. 4d we collated results of low and high field mobility values obtained from several LCs. The horizontal dotted lines denote the values obtained for the cubic lattice representing the standard GDM model. It is interesting that only LC ix (*L* = 90 *nm*; 〈*P*_*Y*_〉 = 43.8 *nm*) exhibits values clearly above that of the cubic lattice. We attribute this to LC ix containing chains that are preferentially aligned in the field direction.

### Section 3–Effects of Energetic Disorder within a Polymer Film like System

**Figure 5:**
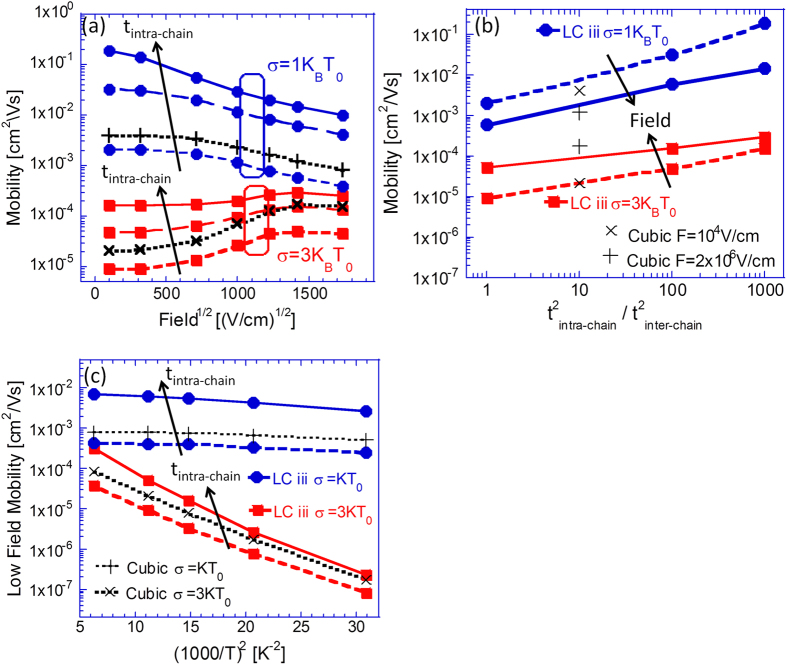
(**a**) Comparison of mobility field dependence curves obtained from simulations implemented with different degrees of energetic disorder and intra-chain transfer integral values. Results obtained from simulations implemented with *σ* = 1 *K*_*B*_*T*_0_ have been multiplied by a factor of 3 for clarity. All results were obtained from simulations implemented on LC iii (*L* = 90 *nm*; 〈*P*_*Y*_〉 = 31 *nm*) at *T* = 300 °*K* with *n* = 6 ⋅ 10^−5^ carriers per chain site in the system. Short dashed lines correspond to data obtained from simulations in which 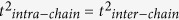, long dashed lines to 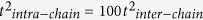 and solid lines to 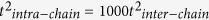. Dotted lines represent the corresponding values obtained from simulations implemented on a cubic lattice. (**b**) Low field *F* = 10^4 ^*Vcm*^−1^ (Dashed lines) and high field *F* = 2 × 10^6 ^*Vcm*^−1^ (Solid lines) mobility values taken from (**a**) as function of the system intra-chain to inter-chain transfer integral ratios. In (Xs) and (+s), mobility values obtained from simulations implemented on a cubic lattice. (**c**) Comparison of mobility temperature dependence curves obtained from simulations implemented with different degrees of energetic disorder and intra-chain transfer integral values. All results were obtained from simulations implemented on LC iii under an applied field of *F* = 10^4 ^*Vcm*^−1^ with *n* = 6 ⋅ 10^−5^ carriers per chain site in the system. Dashed lines correspond to data obtained from simulations in which 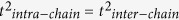 and solid lines to 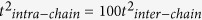. Dotted lines represent the corresponding values obtained from simulations implemented on a cubic lattice.

To attain insight regarding the combined influence of the amorphous polymer film like morphology and energetic disorder, simulations were implemented on LC iii (*L* = 90 *nm*; 〈*P*_*Y*_〉 = 31 *nm*) for different degrees of energetic disorder and intra-chain transfer integral values. Mobility field dependence curves and temperature dependence curves obtained from these simulations are presented in Fig. 5a,c, respectively.

Observing Fig. 5a, the mobilities’ values enhancement and the concomitant decrease of the field dependence curve slopes can be seen to be augmented as the system’s intra-chain transfer integral values are raised (where the usual increase of the system’s mobility and its field dependence slope shift from positive to negative due to the reduction of the system energetic disorder relative to *K*_*B*_*T* can be observed as well^11^). Importantly, the effect of rapid on-chain carrier propagation on the mobility values can be observed to increase as the energetic disorder in the system is reduced. Figure 5b illustrates the effect, where mobility values obtained from both low field *F* = 10^4 ^*Vcm*^−1^ and high field *F* = 2 × 10^6 ^*Vcm*^−1^ simulations are presented as function of the systems’ ratio of intra-chain to inter-chain transfer integrals. Notably, the described effects seem to correlate with the more subtle effect observed in Fig. 5c, in which the mobility temperature dependence curve slopes demonstrate a slight negative shift (larger activation energy) with the increase of the intra-chain transfer integrals. We thus find that, as in previous sections, the rapid on-chain propagation enhances the mobility and alters the field dependence. However, the presence of disorder suppresses the enhancement from ~100 for *σ* = 1 *K*_*B*_*T*_0_ to ~10 for *σ* = 3 *K*_*B*_*T*_0_.

### Section 4–Effects of the Polymers’ Conjugation Segments’ Lengths and of Polaron Transport within a Polymer Film like Morphology

A final feature which was investigated in the context of polymer morphologies is the system’s polymer chains’ conjugation segments’ lengths (i.e. the extent of delocalization of the system’s electronic states). To investigate it, every chain site in the manufactured LCs was defined to represent a chain conjugate segment of constant length *L*_*C*_. The distance associated with hopping between consecutive on-chain sites was, hence, defined to be *L*_*C*_ and the concomitant field related energetic drop was taken to be 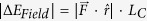, 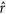 being the unit vector in the hopping direction.

**Figure 6:**
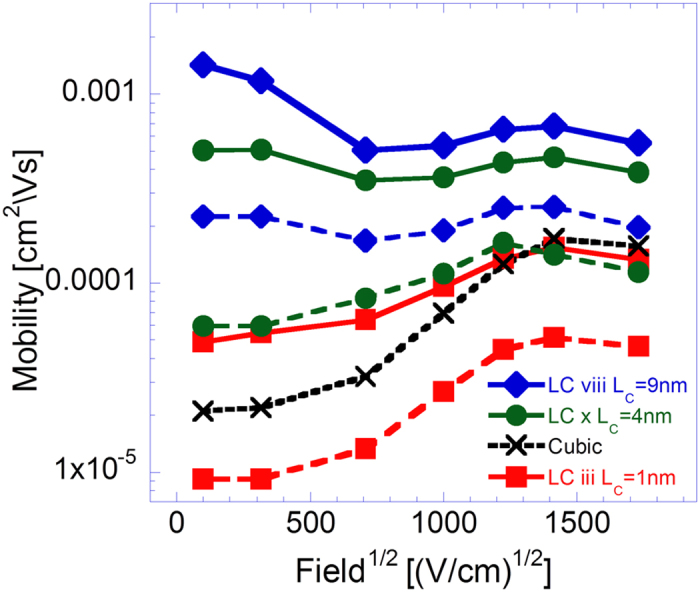
Mobility field dependence curves obtained from simulations implemented on LCs iii (*L* = 90 *nm*), x (*L* = 22 *nm*) and viii (*L* = 10 *nm*) with effective conjugation lengths *L*_*C*_ = 1 *nm*, *L*_*C*_ = 4 *nm* and *L*_*C*_ = 9 *nm*, respectively, and *σ* = 3 *K*_*B*_*T*_0_ (*T*_0_ = 300 °*K*). All figure results obtained from simulations in which 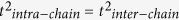 are represented by dashed lines and those in which 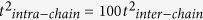 by solid lines. Additionally, all results were obtained from simulations run at temperature *T* = 300 °*K* and *n* = 6 ⋅ 10^−5^ carriers per chain site in the system.

Figure 6 presents the mobility field dependence curves obtained from these simulations, implemented on LCs iii, x and viii, and for which the polymer chains’ conjugation segments’ lengths were set to *L*_*C*_ = 1 *nm* (squares), *L*_*C*_ = 4 *nm* (circles) and *L*_*C*_ = 9 *nm* (diamonds), respectively. All three LCs were constructed such that their respective chains’ lengths (i.e. the number of sites in a chain times *L*_*C*_) and respective average projections on the system axes (i.e. the utilized LC’s average projections times *L*_*C*_) were to a good approximation equal. Observing Fig. 6, the increase of the system’s conjugated segments’ is found to lead to a substantial rise of the system mobility, with the enhancement transcending a factor of 20 at low fields for simulations implemented with a conjugation segment length *L*_*C*_ = 9 *nm*. In addition, as *L*_*C*_ is increased the mobility field dependence curve slopes are found to become more negatively inclined up to moderate fields. At high fields, however, the curve slopes appear to converge. Here, both effects seem to arise from the elongation of the intra-chain hopping distances and the increase of the concomitant energetic drops due to the increase of *L*_*C*_. In addition the observed mobility’s field dependence seems to result from the independence of the hopping distances corresponding to inter-chain hops of *L*_*C*_, leading to the field’s influence on the inter-chain hops to become sufficiently substantial only at higher fields. We note that the field dependence curves obtained from simulations incorporating elongated conjugation segments and rapid on-chain carrier propagation differ substantially from the commonly experimentally observed Poole-Frenkel type field dependence.

**Figure 7:**
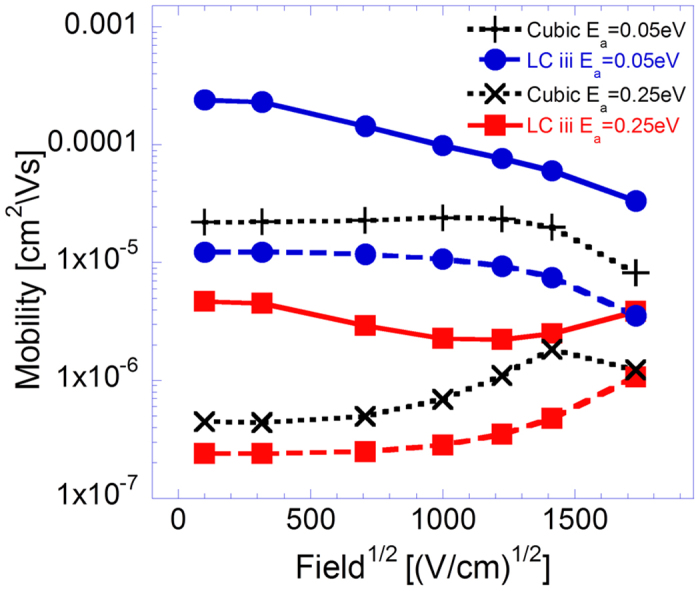
Mobility field dependence curves obtained from polaron transport simulations implemented on LC iii with *σ* = 0. Results obtained from simulations implemented on a cubic lattice are presented for reference. All figure results obtained from simulations in which 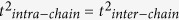 are represented by dashed lines and those in which 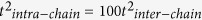 by solid lines. Additionally, all results were obtained from simulations run at temperature *T* = 300 °*K* and *n* = 6 ⋅ 10^−5^ carriers per chain site in the system.

To examine the effect of incorporating polaron transport within an amorphous polymer film like morphology, transport simulations in which carrier transfer rates were determined via Eq. (3) were implemented as well. Results obtained from simulations implemented on LC iii with polaron activation energies *E*_*a*_ = 0.05 *eV* and *E*_*a*_ = 0.25 *eV* are presented in Fig. 7. Here we assume a highly ordered film and idealize it by setting *σ* = 0 3. The data presented in Fig. 7 exhibits similar trends to those observed in Figs 2 and 5, namely the system’s mobility values are found to increase as rapid on-chain carrier propagation is incorporated into the simulations whereas the concomitant curve slopes are found to decrease. However, the curve slopes’ inclination to decrease seems to be here more pronounced, despite the systems’ activation energies being larger than those of the corresponding systems which mobility field dependence curves are presented in Fig. 5a (e.g. the mobility activation energy corresponding to *σ* = 3 *K*_*B*_*T*_0_ is by all measures equal to or smaller than 0.2 *eV*^32^ whereas the activation energy of the corresponding curves in Fig. 7 is *E*_*a*_ = 0.25 *eV*). This can, nevertheless, be partially attributed to the inherent weaker mobility field dependence characteristic of the polaron model (which can be observed by comparing the results obtained from the simulations implemented on a cubic lattice)^33^. In addition, since the applied field facilitates polaronic charge transfer only in the direction of the field, which within a polymer film like system is often a nonexistent possibility, the field’s influence on the system’s mobility within the polaron model is curbed further.

Notably, the results obtained from the polaron simulations in which rapid on-chain carrier propagation was incorporated do not concur with the often experimentally observed Poole-Frenkel type mobility field dependence, thereby differing from the polymer chains’ model output put forward in ref. 3 as well. We attribute this difference between the two model outputs to the model assumption made in ref. 3 that inter-chain hops always occur in the direction of the field force and can occur from any chain site in the system. This is in difference with the present model within which inter-chain hops can occur in all system directions and within which inter-chain hopping possibilities in the direction of the field force are often unavailable.

## Discussion

The influence of material morphology on the transport properties of disordered polymer films has become a subject of increased interest due to the emergence of morphologically disordered polymer films exhibiting mobility values in the higher ranges corresponding to organic semiconducting crystals. Motivated by this emergence, the presented study’s main objective was to provide a general analysis of the effects of chain morphology in amorphous polymer films on these systems transport and thermoelectric effect related properties. We chose to, mainly, study isotropic films where no preferential chain alignment is induced. The analysis was performed through dissecting the morphology of these systems into its principle attributes, and examining each of these attributes’ influence on the system’s charge transport and thermoelectric properties. The influence of energetic disorder and the charge transport mechanism on the transport properties of an amorphous polymer film like system was investigated as well.

In previous publications^13^,^14^ we claimed that the Gaussian Disorder Model (GDM) contains many free parameters and hence, one needs to combine transport and thermoelectricity measurements to less ambiguously determine the material parameters. In that sense, adding the polymer morphology is counterproductive as it introduces several new parameters. However, although outside the scope of the authors expertise, we believe that in a real film the parameters are dependent and molecular calculations^34^ could reduce the parameter space significantly. As such inter-dependencies are not available to us at present we outline the main findings below assuming the various parameters are independent:

1. The most influential parameters on the charge carrier mobility are the energetic disorder, the average number of NN (~film’s density), the carrier delocalization (conjugation) length, and the on-chain transport. The preferential on-chain transport is significant mainly for energetic disorder below 3 K_B_T_0_ and its effect is enhanced when some alignment in the field direction is introduced (LC ix in Fig. 4).
2. The neighboring statistics, which is the most fundamental morphological attribute, was found to additionally affect the system’s thermoelectric properties. Namely, lowering the number of NNs in the system was found to suppress the carrier heating effect (Fig. 3a) and shift the carrier transport energy into higher energies (Fig. 3b). Here, we attributed the former effect to the applied field’s decreasing influence on the carrier trajectories as the system’s neighboring statistics is lowered. Similarly, we ascribed the rise of the transport energy to the decreasing influence of the system’s energetic disorder on the carrier trajectories, resulting from the lowering of the system’s neighboring statistics as well.
3. Incorporating rapid on-chain carrier propagation into the studied systems was found to enhance the measured carrier mobility values. The effect was found importantly, nevertheless, to depend on the extent of energetic disorder in the system (and to a lesser degree on the system’s temperature) (Fig. 5). Namely, the smaller the ratio between the enhancement factor corresponding to the intra-chain hopping rates (due to the corresponding augmented intra-chain transfer integrals), and the system’s degree of energetic disorder, i.e. 
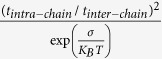
, the more likely was the on-chain propagation to be hampered by on-chain energetic barriers.
4. Diluting the film (lower NN), enhancing the on-chain transport, and implementing a long conjugation length reduces the slope of the mobility as a function of the square root of the electric field. Comparing attained curve slopes with those obtained from the standard GDM model in which the slope depends on the system energetic disorder through 
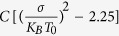
 with C being a material constant^35^, we find that reducing the number of NN to ~2.5 (Fig. 2a) and elongating the conjugation to 9 nm (Fig. 6) makes the dependence look as if the disorder was reduced from 3 K_B_T_0_ to 1 K_B_T_0_. Also, enhancing the on-chain transport by a factor of 1000 reduces the apparent disorder to 2.4 K_B_T_0_.
5. The average chain projections on the system axes, the chains’ length and the ratio between the two *L*/〈*P*_*i*_〉 were found to only modestly influence the system’s carrier mobility (Fig. 4). Namely, the rigidity of the polymer by itself does not affect much the transport properties. In real systems rigid polymers may be better ordered and contain longer conjugation lengths. However, as mentioned above, in this study we looked into each effect separately.
6. Implementing polaron transport simulations within a polymer film like morphology was found to lead to more negatively inclined mobility field dependence curve slopes (Fig. 7) than those corresponding to bare carriers propagating in an energetically disordered medium (Fig. 5a). Here, we attributed the effect, first, to the more inherently mild mobility field dependence found in polaron dominated transport (due to the absence of carrier heating). Second, the fact that within the polaron model the applied field facilitates only charge transfer in the direction of the field force, an often unavailable possibility within the amorphous polymer film like system.

An estimation of a possible mobility enhancement factor that can arise from a combined presence of the described attributes shows a substantial enhancement can be yielded. Namely, mobility values of a ‘standard’ system (i.e. in the range of *μ* ≈ 10^−4 ^*cm*^2^*V*^−1^*s*^−1^) can be enhanced by more than five orders of magnitude, given that its energetic disorder is reduced from *σ* = 3 *K*_*B*_*T* to *σ* = *K*_*B*_*T* (up to almost 2 orders of magnitude increase as shown in Fig. 5a), the film is made denser (up to a factor of 5 increase by making a moderately dense system more dense as shown in Fig. 2a), rapid on-chain carrier propagation is incorporated into it (increase of up to 2 orders of magnitude as shown in Fig. 5a), its chain rigidity increased (20–30% increase as shown in Fig. 4), and its chains’ conjugation segments’ length is increased as well (up to an order of magnitude increase as shown in Fig. 6). Aligning the chains in the field direction would allow reaching similar mobility values even if the attributes just mentioned are not all fully modified (improved).

Given that the mobility field dependence curves arising from the presence of several of the system attributes examined in this study do not concur with the commonly experimental dependences found in disordered organic semiconductors, we envision that measuring this system feature in the emerging aforementioned set of ‘high mobility’ materials^4^ can be particularly informing. Here it should be additionally noted, nevertheless, that while disordered organic semiconductors usually exhibit Poole-Frenkel type behaviors, examples of other types of field dependence forms have been found to occur in disordered polymer systems^11^,^36^,^37^. Indeed, in these systems measured results were discerned through the incorporation of spatial disorder into the GDM framework^11^,^38^ which in similarity with the framework presented here, can lead to the creation of fast local carrier routes in the system as well as to the decrease of the system’s neighboring statistics.

To conclude, the presented study’s main objectives were to provide wide and comprehensive examinations of the influence of the morphology of amorphous polymer films on these system’s transport and thermoelectric properties. By aiming to yield new understanding regarding these systems’ structure-function relations we hope that the findings of the presented study will contribute to the development and improvement of new and existing design strategies for next generation polymer based semiconductors.

## Methods

### Construction of the artificial chain lattice configurations

Construction of chain configurations started with the random placement of chains within the cubic base lattice. Chain movement within the lattice was then simulated. Each simulation step began with the random selection of one of the system’s chain ends. One of the selected end’s unoccupied nearest neighboring sites was then chosen randomly as well and the selected chain’s remaining unselected end was transferred into it given that the inequality 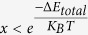 was satisfied. Here, Δ*E*_*total*_ is the change in the system’s energy corresponding to the considered step, *x* a randomly generated number between 0 and 1, *K*_*B*_ Boltzmann’s constant and *T* the simulation temperature. The system’s energy *E*_*total*_ was determined by summing up all energy contributions arising from interfaces between sites affiliated with different chains 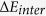 as well as intra-chain site interfaces 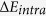 (given that the sites were not sequentially located on the chain). To limit chain self-entanglement in the manufactured LCs, intra-chain sites’ interfaces were assigned higher values than inter-chain interfaces, namely 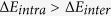. The chain entanglement (or rigidity) was controlled through the LCs’ chain projection (*P*_*i*_) and hence, energies 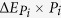 were subtracted from the system energy *E*_*total*_.

All simulations were initially run at high temperatures *T* = 10^4 ^*K* to facilitate chain mixing, with 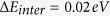, 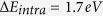 and 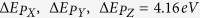. To ensure spatial homogeneity of the constructed LCs, the system’s energy *E*_*total*_ and the chains’ projection distributions *P*_*X*_, *P*_*Y*_, *P*_*Z*_ were monitored during the runs, where upon reaching steady-state, the system’s temperature was cooled to *T* = 150 °*K* (low enough to allow the attainment of homogeneous LCs, but sufficiently high for simulations to run effectively). Concomitantly, the energies 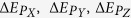 were set to 0.1 *eV*, 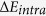 to 0.04 *eV* and 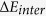 was kept at 0.02 *eV*. Simulations were then allowed to run once again until steady-state or near-steady-state (if convergence was exceptionally slow) was reached.

### Simulations of Carrier Transport

Simulation dynamics were governed by stochastic charge carrier hopping events between chain-sites, where the occupancy of a single site by two carriers was prohibited due to the large Coulomb energy associated with closely placed carriers. Carrier hopping rates were determined via either the Miller-Abrahams^23^ or Marcus-theory^24^ expressions, Eqs (2) and (3) respectively.


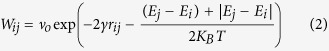



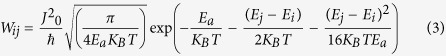


In Eqs (2 and 3) which express the hopping rates between sites *i* and *j*, *E*_*i*_ and *E*_*j*_ represent the site energies, *r*_*ij*_ the effective distance between the sites and *K*_*B*_ Boltzmann’s constant. *ν*_0_ in Eq. (2) represents the hopping attempt rate coefficient and *E*_*a*_ in Eq. (3) represents the polaron activation energy. The transfer integral *J* corresponding to sites *i* and *j*, appearing in Eq. (3), was taken to be 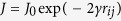 where *γ*, also appearing in Eq. (2), is the carrier inverse localization length (taken to equal through out the study 5 [1/nm]) and *J*_0_ is a constant pre-factor.

Simulations began with the random placement of charge carriers within the LC chain sites. In the simulations’ dynamics, every hopping event’s time and destination were determined by the drawing of a random dwell time via Eq. (4) for each of the 125 nearest (base) lattice sites to a selected carrier, and picking the site associated with the shortest drawn dwell time,


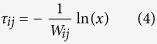


where *x* in Eq. (4) is a randomly generated number between 0 and 1. Simulation runs were initially allowed to reach steady state, upon which variables such as the system’s carrier mobility, transport energy and effective temperature were computed. Further information regarding the implementation of standard GDM transport simulations can be found in refs 21,22.

### Distance reduction rule

**Figure 8:**
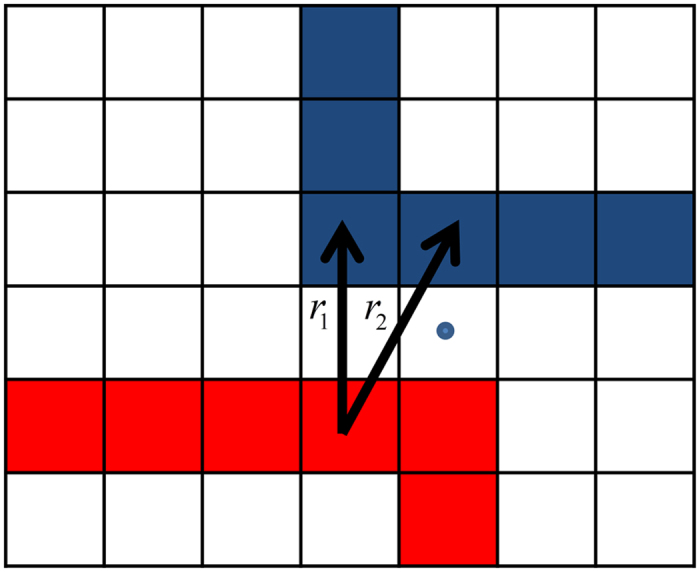
Illustration of the utilized distance reduction rule: given two different polymer chains located in proximity, the distances *r*_1_ = 2*a* and 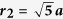 are reduced to the effective distances *r*_1_ = *a* and 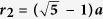.

The rule was employed within the transport simulations to effectively increase the neighboring statics of the studied LCs. It was exercised for sites corresponding to different chains given that between the chain-sites resided at least one empty site which center was distanced less or equal to (*a*)/(2) from the line connecting the chain sites’ centers. If the condition was fulfilled, the distance between the chain sites was effectively reduced by *a*. Figure 8 exemplifies the rule’s application, where the effective distances *r*_1_and *r*_2_ are reduced to *r*_1_ = *a* and 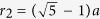 from *r*_1_ = 2*a* and 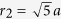, respectively.

## Additional Information

**How to cite this article**: Mendels, D. and Tessler, N. A Comprehensive study of the Effects of Chain Morphology on the Transport Properties of Amorphous Polymer Films. *Sci. Rep.* **6**, 29092; doi: 10.1038/srep29092 (2016).

## Figures and Tables

**Table 1:** List of the 3D lattice configurations (LCs) for which transport simulation results are presented in this paper.

Lattice configuration	Base lattice edge length	Chains’ length	Average chain projection on field axis	Average number of NN	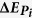	Presented in Figures
Cubic	*N* = 100 *nm*			6		2, 3, 4, 5, 6, 7
i	*N* = 140 *nm*	*L* = 90 *nm*	〈*P*_*Y*_〉 = 31.2 *nm*	2.4	0.1 eV	2
ii	*N* = 140 *nm*	*L* = 90 *nm*	〈*P*_*Y*_〉 = 31 *nm*	2.88	0.1 eV	2,3
iii	*N* = 140 *nm*	*L* = 90 *nm*	〈*P*_*Y*_〉 = 31 *nm*	4	0.1 eV	2, 3, 4, 5, 6, 7
iv	*N* = 140 *nm*	*L* = 90 *nm*	〈*P*_*Y*_〉 = 14.3 *nm*	3.9	0 eV	4
v	*N* = 180 *nm*	*L* = 140 *nm*	〈*P*_*Y*_〉 = 19.6 *nm*	3.95	0 eV	4
vi	*N* = 180 *nm*	*L* = 140 *nm*	〈*P*_*Y*_〉 = 49 *nm*	3.95	0.1 eV	4
vii	*N* = 100 *nm*	*L* = 40 *nm*	〈*P*_*Y*_〉 = 14 *nm*	3.95	0.03 eV	4
viii	*N* = 100 *nm*	*L* = 10 *nm*	〈*P*_*Y*_〉 = 3.45 *nm*	3.86	0 eV	4, 6
ix	*N* = 140 *nm*	*L* = 90 *nm*	〈*P*_*Y*_〉 = 43.8 *nm*	4.12	0.1 eV	4
x	*N* = 100 *nm*	*L* = 22 *nm*	〈*P*_*Y*_〉 = 8 *nm*	4	0.1 eV	6

LC ix was intentionally stopped before reaching the isotropic equilibrium chain distribution.

## References

[b1] Noriega R. . A general relationship between disorder, aggregation and charge transport in conjugated polymers. Nat. Mater. 12, 1038–1044 (2013). 23913173 10.1038/nmat3722

[b2] Carbone P. & Troisi A. Charge Diffusion in Semiconducting Polymers: Analytical Relation between Polymer Rigidity and Time Scales for Intrachain and Interchain Hopping. J. Phys. Chem. Lett. 5, 2637–2641 (2014). 26277956 10.1021/jz501220g

[b3] Noriega R., Salleo A. & Spakowitz A. J. Chain conformations dictate multiscale charge transport phenomena in disordered semiconducting polymers. Proc. Natl. Acad. Sci. USA 110, 16315–16320 (2013). 24062459 10.1073/pnas.1307158110 PMC3799354

[b4] Venkateshvaran D. . Approaching disorder-free transport in high-mobility conjugated polymers. Nature 515, 384–388 (2014). 25383522 10.1038/nature13854

[b5] D’Innocenzo V., Luzio A., Petrozza A., Fazzi D. & Caironi M. Nature of Charge Carriers in a High Electron Mobility Naphthalenediimide Based Semiconducting Copolymer. Adv. Funct. Mater. 24, 5584–5593 (2014).

[b6] Zhang X. . Molecular origin of high field-effect mobility in an indacenodithiophene-benzothiadiazole copolymer. Nat. Commun. 4, (2013). 10.1038/ncomms3238 23900027

[b7] Bassler H. & Kohler A. Charge Transport in Organic Semiconductors. Top. Curr. Chem. 312, 1–65 (2012). 21972021 10.1007/128_2011_218

[b8] Street R. a., Northrup J. E. & Salleo A. Transport in polycrystalline polymer thin-film transistors. Phys. Rev. B 71, 165202 (2005).

[b9] Mollinger S. A., Krajina B. A., Noriega R., Salleo A. & Spakowitz A. J. Percolation, Tie-Molecules, and the Microstructural Determinants of Charge Transport in Semicrystalline Conjugated Polymers. ACS Macro Lett. 4, 708–712 (2015). 10.1021/acsmacrolett.5b00314 35596492

[b10] Liu T. & Troisi A. Understanding the Microscopic Origin of the Very High Charge Mobility in PBTTT: Tolerance of Thermal Disorder. Adv. Funct. Mater. 24, 925–933 (2014).

[b11] Bassler H. Charge Transport in Disordered Organic Photoconductors. phys. stat. sol. 175, 15–56 (1993).

[b12] Yu. N. Gartstein & Conwell E. M.. High-field hopping mobility in molecular systems with spatially correlated energetic disorder. Chem. Phys. Lett. 245, 351–358 (1995).

[b13] Mendels D. & Tessler N. Thermoelectricity in Disordered Organic Semiconductors under the Premise of the Gaussian Disorder Model and Its Variants. J. Phys. Chem. Lett. 5, 3247–3253 (2014). 26276340 10.1021/jz5016058

[b14] Mendels D. & Tessler N. Field dependent thermoelectric properties of organic semiconductors–A tool to determine the nature of charge transport in materials exhibiting thermally activated transport. J. Appl. Phys. 117, 105502 (2015).

[b15] Mladenović M. & Vukmirović N. Charge Carrier Localization and Transport in Organic Semiconductors: Insights from Atomistic Multiscale Simulations. Adv. Funct. Mater. 25, 1915–1932 (2015).

[b16] Kron A. K. The Monte Carlo Method in Statistical Calculations of Macromolecules. Pol. Sci. USSR 7, 1228–1234 (1965).

[b17] Wall F. T. & Mandel F. Macromolecular dimensions obtained by an efficient Monte Carlo method without sample attrition. J. Chem. Phys. 63, 4592–4595 (1975).

[b18] Frost J. M., Cheynis F., Tuladhar S. M. & Nelson J. Influence of Polymer-Blend Morphology on Charge Transport and Photocurrent Generation in Donor–Acceptor Polymer Blends. Nano Lett. 6, 1674–1681 (2006). 16895355 10.1021/nl0608386

[b19] Qin T. & Troisi A. Relation between structure and electronic properties of amorphous MEH-PPV polymers. J. Am. Chem. Soc. 135, 11247–11256 (2013). 23829780 10.1021/ja404385y

[b20] Baschnagel J., Wittmer J. P. & Meyer H. Monte Carlo simulation of polymers: coarse-grained models. Computational Soft Matter: From Synthetic Polymers to Proteins (NIC Series vol. 23) ed. Attig N. . 83–140 (2004). (Preprint cond-mat/0407717).

[b21] Mendels D. & Tessler N. Drift and Diffusion in Disordered Organic Semiconductors: The Role of Charge Density and Charge Energy Transport. J. Phys. Chem. C 117, 3287–3293 (2013).

[b22] Mendels D. & Tessler N. The Topology of Hopping in the Energy Domain of Systems with Rapidly Decaying Density of States. J. Phys. Chem. C 117, 24740–24745 (2013).

[b23] Miller A. & Abrahams E.. Impurity Conduction at Low Concentrations. Phys. Rev. 120, 745 (1960).

[b24] Marcus R. A. Chemical and Electrochemical Electron-Transfer Theory. Annu. Rev. Phys. Chem. 15, 155–196 (1964).

[b25] Fornari R. P., Aragó J. & Troisi A. A very general rate expression for charge hopping in semiconducting polymers. J. Chem. Phys. 142, 184105 (2015). 25978881 10.1063/1.4920945

[b26] Preezant Y. & Tessler N. Carrier heating in disordered organic semiconductors. Phys. Rev. B 74, 235202 (2006).

[b27] Jansson F., Baranovskii S. D., Sliaužys G., Österbacka R. & Thomas P. Effective temperature for hopping transport in a Gaussian DOS. Phys. Status Solidi 5, 722–724 (2008).

[b28] Marianer S. & Shklovskii B. I. Effective temperature of hopping electrons in a strong electric field. Phys. Rev. B 46, 100–103 (1992). 10.1103/physrevb.46.13100 10003349

[b29] Overhof H. Thermopower Calculation for Variable Range Hopping - Application to a-Si. phys. stat. sol. 67, 709–714 (1975).

[b30] Mott N. F. Electronic processes in non-crystalline materials (Clarendon Press, 1979).

[b31] Onsager L. Irreversible processes. Phys. Rev. 37, 237–241 (1931).

[b32] Hartenstein B. & Bässler. H. Transport energy for hopping in a Gaussian density of states distribution. J. Non-Cryst. Solids 190, 112 (1995).

[b33] Tessler N., Preezant Y., Rappaport N. & Roichman Y. Charge Transport in Disordered Organic Materials and Its Relevance to Thin-Film Devices: A Tutorial Review. Adv. Mat. 21, 2741–2761 (2009).

[b34] Do H. & Troisi A. Developing accurate molecular mechanics force fields for conjugated molecular systems. Phys. Chem. Chem. Phys. 17, 25123–25132 (2015). 26349916 10.1039/c5cp04328j

[b35] Van Der Auweraer B. M., De Schryver F. C., Borsenberger P. M. & Bassler H. Disorder in Charge Transport in Doped Polymers. Adv. Mat. 6, 199–213 (1994).

[b36] Mozer A. J. . Charge carrier mobility in regioregular poly(3-hexylthiophene) probed by transient conductivity techniques: A comparative study. Phys. Rev. B 71, 035214 (2005).

[b37] Mozer A. J. & Sariciftci N. S. Negative electric field dependence of charge carrier drift mobility in conjugated, semiconducting polymers. Chem. Phys. Lett. 389, 438–442 (2004).

[b38] Gartstein Y. N. & Conwell E. M. High-field hopping mobility in disordered molecular solids: A Monte Carlo study of off-diagonal disorder effects. J. Chem. Phys. 100, 9175 (1994).

